# Supplementary material for: Identification of photoperiod-induced specific miRNAs in the adrenal glands of Sunite sheep (Ovis aries)
Source: Front Vet Sci. 2022 Jul 22;9:888207. doi: 10.3389/fvets.2022.888207 (PMC9354845; doi:10.3389/fvets.2022.888207)
Supplement: Supplementary file 1 [file Data_Sheet_1.ZIP › Supplementary file/miRDeep2 file.zip!/miRDeep2 file/Novel_Structure/SPLP42A2/Novel_156.pdf]

Provisional ID	:	NC_019458.2_1514
Score total	:	52.5
Score for star read(s)	:	3.9
Score for read counts	:	45
Score for mfe	:	2
Score for randfold	:	1.6
Score for cons. seed	:	
Total read count	:	100
Mature read count	:	98
Loop read count	:	0
Star read count	:	2

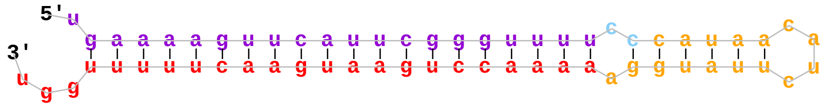

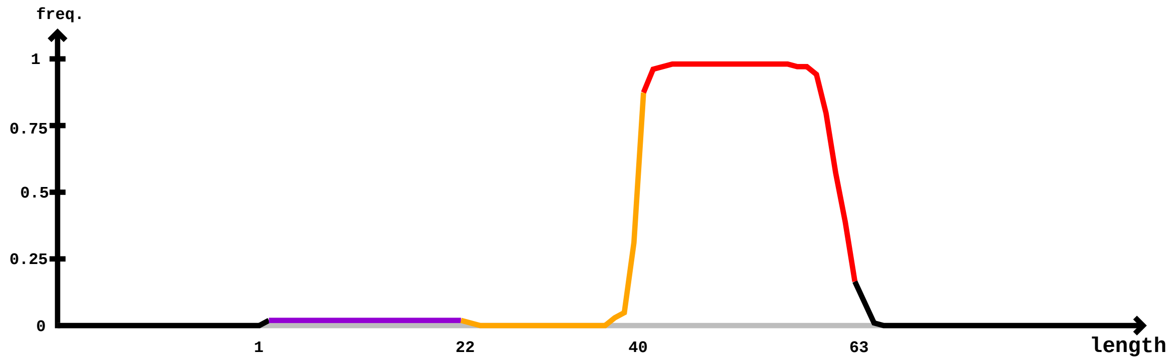

Star Mature

```
5'- gaaagaaguauugcgucggcugaaaaguucauucggguuuucccauaacaucuuauggaaaaaccugaaugaacuuuuuggucaacccaauauaaggaggagacaaagaga  -3'  obs
    gaaagaaguauugcgucggcugaaaaguucauucggguuuucccauaacaucuuauggaaaaaccugaaugaacuuuuuggucaacccaauauaaggaggagacaaagaga        exp
    .......((((((.((.((((((((((((((((((((((((.((((((....)))))).)))))))))))))))))))))))).)).))))))..................   reads    mm    sample
    ....................ugaaaaguucauucggguuuu......................................................................   2        0     seq
    ........................................................ggaaaaaccugaaugaacuuu..................................   1        0     seq
    ........................................................Ugaaaaaccugaaugaacuuuu.................................   1        1     seq
    ........................................................ggaaaaaccugaaugaacuuuu.................................   1        0     seq
    .........................................................gaaaaaccugaaugaacuuC..................................   1        1     seq
    .........................................................gaaaaaccAgaaugaacuuuu.................................   1        1     seq
    ..........................................................aaaaaccugaaugaaUu....................................   1        1     seq
    ..........................................................aaaaaccAgaaugaacuuuu.................................   10       1     seq
    ..........................................................aaaaaccugaaGgaacuuuu.................................   1        1     seq
    ..........................................................aaaaaccugaaugaacuuuuC................................   1        1     seq
    ..........................................................Caaaaccugaaugaacuuuuu................................   1        1     seq
    ..........................................................aaaaaccugaaGgaacuuuuug...............................   12       1     seq
    ..........................................................aaaaaccugaaugaacuuuCug...............................   1        1     seq
    ...........................................................aaaaccAgaaugaacuuu..................................   1        1     seq
    ...........................................................aaaaccAgaaugaacuuuu.................................   1        1     seq
    ...........................................................aaaaccAgaaugaacuuuuu................................   7        1     seq
    ...........................................................Uaaaccugaaugaacuuuuu................................   1        1     seq
    ...........................................................aaaaccugaaugaacuuuuu................................   13       0     seq
    ...........................................................aaaaccugaaugaacuuuuuA...............................   1        1     seq
    ...........................................................Gaaaccugaaugaacuuuuug...............................   2        1     seq
    ...........................................................aaaGccugaaugaacuuuuug...............................   1        1     seq
    ...........................................................aaaaccGgaaugaacuuuuugg..............................   1        1     seq
    ...........................................................aaaaccugaaugaacCuuuugg..............................   7        1     seq
    ...........................................................aaaaccugaaugaacuuuuugU..............................   7        1     seq
    ...........................................................aaaaccugaaugaGcuuuuugg..............................   1        1     seq
    ...........................................................aaaacUugaaugaacuuuuugg..............................   1        1     seq
    ...........................................................aaaaUcugaaugaacuuuuuggu.............................   14       1     seq
    ............................................................aaaccugaaugaacuuuuug...............................   1        0     seq
    ............................................................aaaccAgaaugaacuuuuug...............................   1        1     seq
    ............................................................aaaccugaauAaacuuuuugg..............................   1        1     seq
    ............................................................aaaccugaGugaacuuuuugg..............................   1        1     seq
    ............................................................aaaccugaaugaacuuuuugU..............................   3        1     seq
    ............................................................Uaaccugaaugaacuuuuugg..............................   1        1     seq
```

```
                    Star                                   Mature
gaaagaaguauugcgucggcugaaaaguucauucggguuuucccauaaacaucuuauggaaaaaccugaaugaacuuuuuggucaacccaauauaaggaggagacaaagaga
.............................................................aaaccugaaugaacuuuuugUu.............................     1     1     seq
..............................................................aacUugaaugaacuuuuuggu.............................     1     1     seq
...............................................................acUugaaugaacuuuuugguca...........................     1     1     seq
```
